# Understanding Tryptase Physiology, Clinical Significance, and Diagnostic Applications: Where Do We Stand?

**DOI:** 10.3390/biom16091360

**Published:** 2026-09-18

**Authors:** Mara De Amici, Ivan Taietti, Riccardo Castagnoli, Grazia Bossi, Simone Foti Randazzese, Alessia Marseglia, Silvia Maria Elena Caimmi, Amelia Licari

**Affiliations:** 1 Immuno-Allergology Laboratory of Clinical Chemistry and Pediatric Clinic, Fondazione IRCCS Policlinico San Matteo, 27100 Pavia, Italy; m.deamici@smatteo.pv.it; 2 Pediatric Unit, Department of Clinical, Surgical, Diagnostic and Pediatric Sciences, University of Pavia, 27100 Pavia, Italy; i.taietti@smatteo.pv.it (I.T.); s.fotirandazzese@smatteo.pv.it (S.F.R.); amelia.licari@unipv.it (A.L.); 3 Pediatric Clinic, Fondazione IRCCS Policlinico San Matteo, 27100 Pavia, Italy; g.bossi@smatteo.pv.it (G.B.); a.marseglia@smatteo.pv.it (A.M.); s.caimmi@smatteo.pv.it (S.M.E.C.)

**Keywords:** tryptase, mast cell activation, anaphylaxis, systemic mastocytosis, hereditary alpha-tryptasemia, baseline serum tryptase, mastocytosis

## Abstract

Tryptase is the principal biomarker of mast cell (MC) burden and activation, routinely used in the diagnosis and management of MC-related disorders. Beyond its established role in confirming anaphylaxis, serum tryptase has gained increasing relevance in the diagnosis of systemic mastocytosis, hereditary α-tryptasemia (HαT), cutaneous mastocytosis, and selected haematologic diseases. Recent advances in MC biology, tryptase genetics, and disease classification have substantially improved the interpretation of baseline and acute (event-related) tryptase measurements. In particular, validation of the “20% + 2” consensus formula for MC activation and the recognition of HαT as a common genetic cause of elevated baseline serum tryptase have refined diagnostic algorithms and risk-stratification strategies. This review summarizes current knowledge on tryptase physiology, laboratory assessment, and clinical significance across multiple disease settings, with particular emphasis on the interpretation of baseline serum tryptase, the diagnostic role of tryptase in anaphylaxis and mastocytosis, and emerging applications in allergy, hematology, and chronic inflammatory disorders. Understanding both the strengths and limitations of serum tryptase measurement is essential for accurate diagnosis, prognostic assessment, and personalized patient management.

## 1. Introduction

Tryptase is the most represented protein within mast cells (MCs) granules and is released immediately into the bloodstream during the activation of MC with both immunoglobulin E (IgE) and non-IgE-mediated mechanisms [1]. It is currently the main MC biomarker available in medical practice [2]. MC tryptase is a tetrameric neutral serine protease with a molecular weight of 134 kDa. The enzyme is made up of four non-covalently bound subunits, and each subunit has one active enzyme site. Two are the main types of MC tryptase, α-tryptase and β-tryptase, both isoforms secreted constitutively as enzymatically inactive proforms, whose continuous efflux determines the baseline serum concentration. Mature β-tryptase, by contrast, is stored in the secretory granules and released on degranulation, and is therefore the form that reflects acute MC activation [3]. Tryptase is the most specific readily available marker of MC involvement, but it is not the most sensitive indicator of anaphylaxis: platelet-activating factor (PAF) and PAF-acetylhydrolase activity track reaction severity more closely.

**Figure 1:**
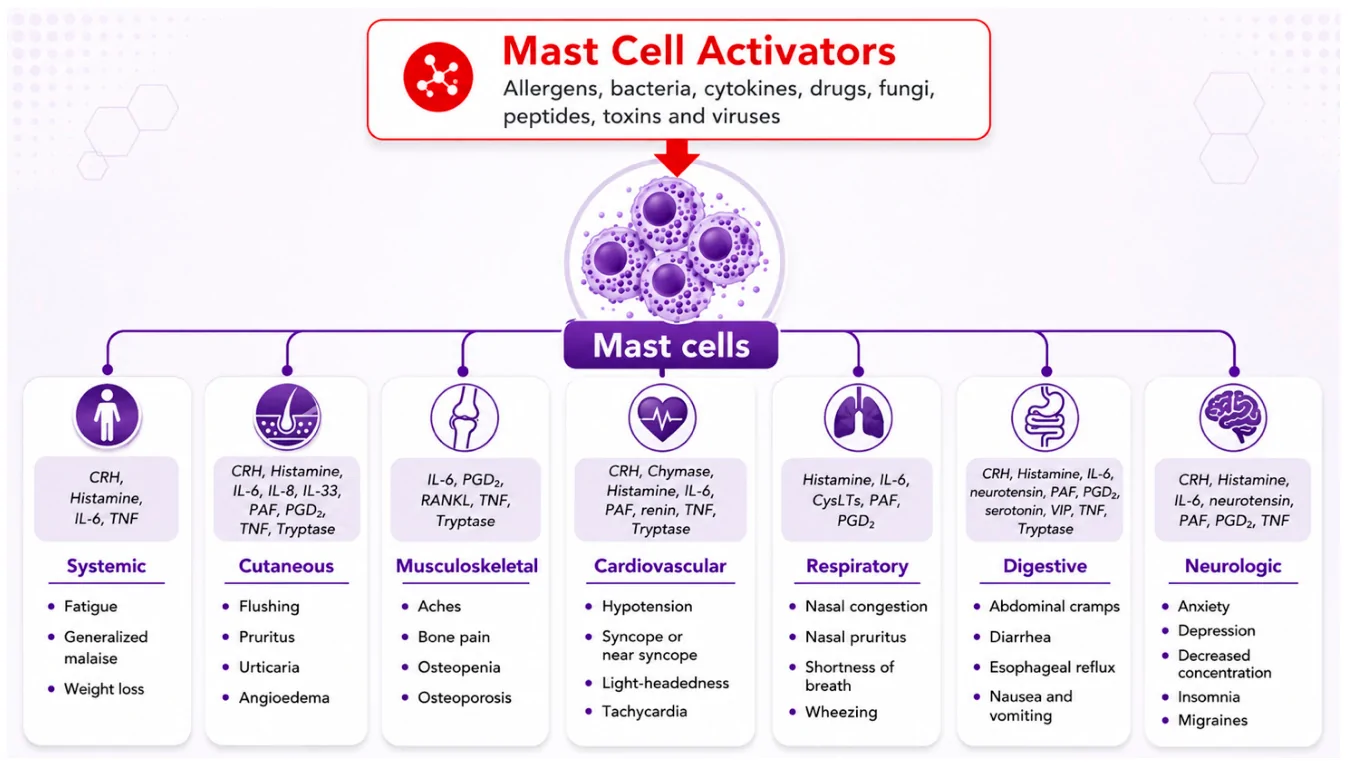
Mast cell activation and release of inflammation mediators. CRH, Corticotropin-releasing hormone. CysLTs, cysteinyl leukotrienes. IL, interleukin. PAF, platelet activating factor. PGD2, prostaglandin D2. RANKL, receptor activator of nuclear factor-κB ligand. TNF, tumoral necrosis factor. VIP, vasoactive intestinal peptide. Figure were created by the authors using Microsoft PowerPoint for Microsoft 365 (Microsoft Corporation, Redmond, WA, USA).

MC tryptase plays an important role in inflammation although the biological function has not been fully clarified. MCs are present in the connective tissues and tend to concentrate particularly along the blood vessels localizing in the lungs, skin, bowel, kidney, liver, synovia, heart, conjunctiva, nasal, respiratory and gastrointestinal (GI) mucosa. MC are involved in the genesis of allergic, hypersensitivity and anaphylactic reactions; today they are considered the activators of acute inflammation. When MC are activated, they secrete very quickly the contents of their granules, heparin (or other sulphated proteoglycans), the biogenic amine histamine and several proteases (e.g., tryptase, chymase and carboxypeptidase A3). All these mediators and chemotactic substances can act in a short time on the contractility of smooth muscles causing bronchoconstriction, on blood vessels modifying their permeability, and on the glandular system determining secretion of hormones (Figure 1) [4,5,6].

The total tryptase test measures the total tryptase circulating in the bloodstream, which includes both proforms and the mature form of the protein and is therefore fundamental in a numerous clinical situations ranging from anaphylactic reactions to hematological disorders and mastocytosis. MC granules contain heparin, histamine and several proteases, which together account for approximately 25% of total MC protein content. Granule-stored β-tryptase is not present as a free active enzyme: it is retained in macromolecular complexes with heparin- and chondroitin sulphate-containing serglycin proteoglycans, an association that is required both for the final processing of the proenzyme and for the stabilization of the catalytically competent tetramer, which dissociates into inactive monomers once released and diluted at neutral pH (see Section 2.2). The extracellular release of these mediators, known as degranulation, may be induced by physical factors, toxins and venoms, endogenous mediators and immune mechanisms, both IgE-dependent and IgE-independent; it is not an all-or-none phenomenon, so that several triggers produce clinically overt mediator-driven symptoms without any measurable rise in histamine or tryptase. During an anaphylactic reaction serum MC tryptase concentration increases. Moreover, increased levels of tryptase can be detected in different hematological diseases and in patients with systemic mastocytosis (SM). The significant release of tryptase from MCs gives a tool, useful to distinguish MC-dependent systemic reactions from other systemic reactions, which can present with similar clinical manifestations. Given the central role of MC in the cascade of allergic reactions, patients with increased MC tryptase levels in serum must be investigated for potential IgE-mediated allergy [7].

## 2. Tryptase Physiology

### 2.1. Tryptase Genes and Isoforms

In humans, the term “tryptase” represents an umbrella for the products of four genes, clustered at chromosome 16p13.3: *TPSG1* (γ-tryptase), *TPSB2* (βII- and βIII-tryptase), *TPSAB1* (α- or βI-tryptase, depending on the allele) and *TPSD1* (δ-tryptase). Only α- and β-tryptases are clinically relevant and clinically measurable: γ-tryptase is membrane-anchored and is not released in soluble form, whereas δ-tryptase is truncated and largely inactive [3,7,8]. The β isoforms (βI, βII, βIII) are fully functional neutral serine proteases and act as the prominent drivers of local inflammation. Mature α-tryptase, by contrast, possesses little or no catalytic activity, owing to amino-acid variations within its substrate-binding domain [8]. Early separation of the native isoforms established that tetramers consist of four homologous rather than mixed subunits, and demonstrated differences between isoforms in the rate and specificity of cleavage of physiological substrates such as high-molecular-mass kininogen and vasoactive intestinal peptide [9]. Because *TPSAB1* and *TPSB2* are inherited as a haplotype and TPSAB1 may carry either allele, healthy individuals differ in their α:β gene ratio, and approximately 30% of the population carries no α-encoding allele at all (ββ:ββ). This constitutional variability is the principal determinant of the wide interindividual range of basal serum tryptase (BST) observed in healthy subjects [10,11,12].

The functional consequence of this genetic architecture is remarkable, and it resolves what would otherwise be a genuine paradox: how a catalytically inert protein comes to be associated with clinically severe disease. The answer lies not in α-tryptase acting alone but in its incorporation into α/β heterotetramers, whose substrate preferences differ from those of β homotetramers. Heterotetramers cleave protease-activated receptor 2 (PAR2) and the epidermal growth factor-like module-containing mucin-like hormone receptor 2 (EMR2), thereby engaging vascular, neuronal and connective tissue targets that β homotetramers do not efficiently reach [10,13,14].

The clinical correlate of this mechanism is well documented and dose dependent. Increased α-tryptase-encoding germline copy number at *TPSAB1* determines a heritable risk of severe anaphylaxis, demonstrated across SM, hymenoptera venom allergy and idiopathic anaphylaxis [15], and is likewise associated with severe food allergic reactions [16]. Beyond anaphylaxis, the phenotype extends across multiple organ systems, encompassing cutaneous, gastrointestinal, autonomic and musculoskeletal manifestations, with wide implications outside allergy practice [14,17]. It is therefore an oversimplification to describe α-tryptase as clinically irrelevant on account of its enzymatic inactivity: it is inactive as a homotetramer, but it is an active modifier of disease severity as a constituent of heterotetramers. The clinical consequences of this trait are considered in detail in Section 2.4.

### 2.2. Biosynthesis, Processing and Granule Storage

Tryptase is mainly synthesized by MCs, although very low amounts originate in basophils, without interfering with total levels [2,18]. Tryptase is synthesized as preprotryptase, from which the signal peptide is removed co-translationally to yield protryptase. Maturation proceeds first through an autocatalytic intermolecular cleavage, strictly dependent on heparin and on the acidic pH of the secretory granule, followed by removal of the residual pro-dipeptide by dipeptidyl peptidase I (cathepsin C) [8,19]. Only cells co-expressing heparin-containing serglycin proteoglycan can complete this pathway, effectively restricting the generation of enzymatically active tryptase to the MC lineage. Mature protomers assemble into a tetramer, with the four active sites facing inwards into a narrow central pore. Access is therefore restricted to small substrates and, critically, the enzyme resists the physiological serine protease inhibitors present in plasma, such as α_1_-antitrypsin and α_2_-macroglobulin. Tetramer integrity depends on ionic interaction with heparin: once secreted and diluted into the interstitium and plasma, the heparin-stabilized complex dissociates into inactive monomers within minutes to hours, so that the immunoreactive tryptase measured in serum is largely a marker of MC events rather than a measure of circulating enzymatic activity [8,19,20,21]. Overall, a substantial proportion of protryptase is not processed to the mature enzyme and is instead secreted constitutively: it is this continuous baseline efflux of α- and β-proforms, and not granule release, that determines BST.

### 2.3. Release Mechanisms and Biological Functions

MC degranulation may be initiated through several distinct routes. The classical pathway is aggregation of the high-affinity IgE receptor FcεRI by allergen-specific IgE. Non-IgE-mediated routes include the complement anaphylatoxins C3a and C5a, IgG-dependent mechanisms, direct activation by physical stimuli, and engagement of MRGPRX2 by cationic drugs and neuropeptides, including neuromuscular blocking agents, fluoroquinolones, vancomycin, opioids and substance P [22]. The route of activation influences which mediators are released and, consequently, which biomarkers become measurable clinically. Of considerable practical importance, degranulation is not an all-or-none phenomenon: depending on the stimulus, the receptor engaged and the intracellular signaling pathway recruited, MCs may undergo degranulation or selectively release newly formed lipid mediators and cytokines with little or no discharge of preformed granule proteins. Several triggers therefore produce clinically overt mediator-driven symptoms without any measurable rise in histamine or tryptase, which is one of the reasons why a normal tryptase value never excludes MC involvement. Once released, tryptase acts as an inflammatory mediator. Through cleavage of PAR2 on endothelial cells, smooth muscle, epithelium and sensory neurons it contributes to vasodilatation, increased vascular permeability, bronchial hyperresponsiveness, neurogenic inflammation and pruritus. It degrades fibrinogen and inactivates fibrinogen-derived procoagulant substrates, contributing to the local anticoagulant milieu of the MC microenvironment; it activates matrix metalloproteinases and pro-urokinase, thereby participating in extracellular matrix remodeling, angiogenesis and fibrosis; and it acts as a chemoattractant for eosinophils and neutrophils. Tryptase further contributes to airway homeostasis, gastrointestinal smooth muscle activity and intestinal transport [7,8]. These activities explain the interest in tryptase not only as a biomarker but also as a therapeutic target, an approach exemplified by the development of allosteric anti-tryptase antibodies [23].

### 2.4. Hereditary α-Tryptasemia

Hereditary α-tryptasemia (HαT) is an autosomal dominant trait caused by increased germline copy number of the α-tryptase-encoding sequence at *TPSAB1* [10]. It is the most common cause of increased BST, affecting approximately 4–6% of Western populations and accounting for a substantially greater proportion of raised tryptase values in clinical practice than clonal MC diseases [11,12,24]. BST rises in a gene-dose-dependent manner: once a TPSAB1 genotype has been established, mean basal concentrations of approximately 15 ± 5 μg/L are reported for an α-tryptase duplication and of approximately 24 ± 6 μg/L for a triplication [10,13]. These genotype-specific mean values must not be confused with the screening thresholds used to select individuals for genotyping (see Section 3.3). The trait is common and many carriers are entirely asymptomatic; nevertheless, it modifies the severity of MC-mediated reactions across several settings (e.g., enrichment among patients with idiopathic anaphylaxis, association with more severe hymenoptera venom-induced reactions, predictor of severe food allergic reactions) [16,25,26]. In patients with SM, it independently predicts severe mediator-related symptoms and cardiovascular anaphylaxis irrespective of MC burden [25,27]. Beyond anaphylaxis, increased α-tryptase gene dosage has been linked with multisystem symptom complexes involving the gastrointestinal tract, the autonomic nervous system, the musculoskeletal system and the skin [10,12,17]. The mechanistic basis is the formation of α/β heterotetramers with distinct substrate specificity rather than any intrinsic activity of α-tryptase itself [13,14].

Practically, tryptase genotyping should be considered in any patient with persistently elevated BST, and specifically in those with idiopathic or severe anaphylaxis, venom allergy or suspected MC disease, since identifying HαT may spare an unnecessary bone marrow examination and refines risk stratification [13,28]. Conversely, HαT and clonal disease are not mutually exclusive and frequently coexist [25,27]. How exactly HαT contributes to the pathogenesis of MC disorders remains incompletely understood, and only a fuller understanding will enable targeted therapeutic approaches for patients with MC disease and coexisting HαT [12].

## 3. Laboratory Assessment of Serum Tryptase

### 3.1. Analytical Methods

Measuring tryptase in biological fluids has become the standard way to gauge both MC numbers and their activation state. The first assay developed for this purpose captured the enzyme using the G5 monoclonal antibody—selective for the mature β form—paired with a goat polyclonal anti-tryptase antibody for detection and was applied to demonstrate tryptase release into the circulation during anaphylactic episodes [29]. Later monoclonal antibodies, notably G4 and B12, recognize both α- and β-protomers and detect proforms as well as mature enzyme, representing the current clinically used assays [7,30].

**Figure 2:**
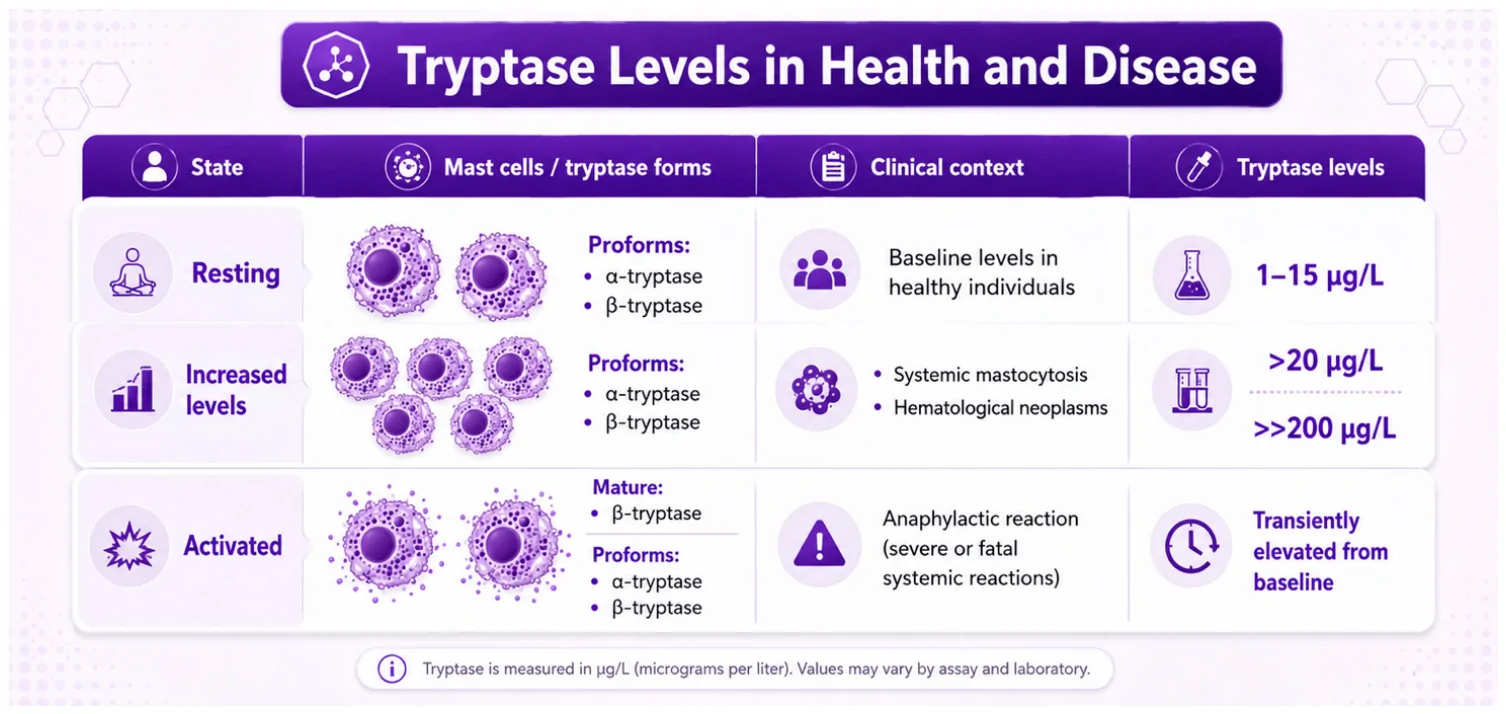
Mast cells and total tryptase (proforms and mature enzyme) in health and disease. Healthy individuals’ baseline values include asymptomatic carriers of hereditary α-tryptasemia. Figure were created by the authors using Microsoft PowerPoint for Microsoft 365 (Microsoft Corporation, Redmond, WA, USA).

In clinical practice the immature monomeric proforms and the mature tetrameric forms cannot be discriminated but can be quantified together as “total tryptase”, most commonly by a fluoroenzyme immunoassay (FEIA; ImmunoCAP, Thermo Fisher Scientific, Uppsala, Sweden), which measures all forms of α- and β-tryptase, with results expressed in μg/L (numerically equivalent to ng/mL). The assay is calibrated against a reference traceable to purified tryptase over a calibrator range of 1–200 μg/L, has a limit of detection below the lowest calibrator (1.0 μg/L), and shows within-assay coefficients of variation of 3–7% and between-assay coefficients of 5–7% across the measuring interval; dilution linearity has been demonstrated between 1 and 198 μg/L, and samples exceeding 200 μg/L require dilution (ImmunoCAP™ Tryptase, Directions for Use 52-5467-EN/05; Phadia AB: Uppsala, Sweden, 2022). Because the great majority of published thresholds derive from this single platform, results obtained with alternative immunoassays are not necessarily interchangeable, and serial determinations in the same patient should ideally be performed in the same laboratory [31,32]. Assays able to quantify α- and β-tryptase separately, or to detect mature β-tryptase specifically, remain confined to research settings—a limitation with direct diagnostic consequences, discussed in Section 3.5 (Figure 2).

### 3.2. Preanalytical Variables and Timing of Sampling

Correct interpretation of a tryptase result depends as much on how and when the sample was obtained as on the value itself. Both serum and plasma (EDTA or heparin) drawn from venous blood are acceptable matrices, and no specific patient preparation is required; unlike histamine, tryptase is not appreciably affected by diet [7] (ImmunoCAP™ Tryptase, Directions for Use 52-5467-EN/05; Phadia AB: Uppsala, Sweden, 2022). Tryptase is a comparatively robust analyte, but storage at room temperature is not recommended: samples may be held at ambient temperature (not exceeding 32 °C) for up to two days where shipping requires it, should be stored at 2–8 °C for up to one week, and otherwise at −20 °C, avoiding repeated freezing and thawing. Marked haemolysis should be avoided, and the assay is unaffected by heparin below 75 IU/mL, by bilirubin and by lipaemia at the concentrations tested (ImmunoCAP™ Tryptase, Directions for Use 52-5467-EN/05; Phadia AB: Uppsala, Sweden, 2022). Post-mortem serum can be assayed, and samples should then be obtained close to the time of death and, where feasible, at intervals thereafter.

Timing is crucial, as following systemic MC activation, serum tryptase rises within 15–30 min, peaks between 15 and 120 min, then declines slowly over the following 3–6 h with a half-life of approximately 2 h, returning to baseline within 24–48 h [33]. The acute sample should therefore be drawn as close as possible to the reaction, overall, within 4 h. The interval between reaction and venipuncture should be recorded, since it is essential to interpretation. A second sample obtained at least 24 h after complete resolution of all clinical symptoms is mandatory to establish the individual baseline and confirm the return to it. Where mastocytosis or HαT is suspected, a further determination 1–2 weeks after the event is advisable (ImmunoCAP™ Tryptase, Directions for Use 52-5467-EN/05; Phadia AB: Uppsala, Sweden, 2022; Canadian Agency for Drugs and Technologies in Health. Retesting intervals for tryptase: rapid review. CADTH Rapid Response Report 2024) [31].

Furthermore, intraindividual variability must be considered. Although BST is a stable constitutional trait, and more stable than most inflammatory markers, serial measurements in the same subject show a median intraindividual coefficient of variation of 15–20% over one year. Recognizing this variability improves the accuracy with which the acute-versus-baseline comparison identifies MC activation and argues for establishing the baseline from more than one determination whenever the clinical stakes are high [24,34].

### 3.3. Reference Intervals

The reference interval for serum tryptase has been significantly revised over the years. For many years the upper reference limit adopted in most laboratories was 11.4 μg/L, a value derived from the distribution of results in a reference population and widely used as the threshold defining “elevated” tryptase [35] (ImmunoCAP™ Tryptase, Directions for Use 52-5467-EN/05; Phadia AB: Uppsala, Sweden, 2022). In 2022 the experts of the European Competence Network on Mastocytosis (ECNM) and of the American Initiative in Mast Cell Diseases concluded that the normal range should be defined in asymptomatic controls, explicitly including individuals with HαT, and calculated over two standard deviations covering the 95% confidence interval. On that basis the normal BST range is now considered to span 1 to 15 μg/L [31], thus avoiding unnecessary referrals, bone marrow examinations and considerable patient anxiety generated by the former, lower threshold. Practically, values between roughly 11 and 15 μg/L should be regarded as an indication for tryptase genotyping rather than as evidence of clonal disease, whereas the diagnostic thresholds embedded in disease-specific criteria remain unchanged [13,36]. Pediatric reference values deserve separate consideration. BST is significantly higher during the first year of life and declines thereafter, being influenced by sex hormones, with lower values in females from puberty until menopause. On this basis it has been proposed that the HαT screening threshold of 8.0 μg/L is appropriate for boys, whereas a lower threshold of 7.0 μg/L may be more suitable for girls from the age of 5 years. These are screening thresholds used to select children for tryptase genotyping, and must not be confused with the genotype-specific mean concentrations reported once a *TPSAB1* duplication or triplication has been confirmed (see Section 2.4) [37].

### 3.4. Limitations of Serum Tryptase Measurement

Several limitations must be addressed, and they define the circumstances in which a tryptase result should not be relied upon. Firstly, commercially available assays quantify total tryptase and do not discriminate between α- and β-forms, nor between proforms and mature enzyme. A single elevated baseline value is therefore intrinsically ambiguous, since it may reflect an increased MC burden, ongoing activation, or increased constitutive transcription [7,32]. The second limitation is limited sensitivity in the acute setting. Tryptase frequently remains within the normal range in localized or mild allergic reactions, and severe food-induced anaphylaxis often occurs without any measurable elevation. Moreover, transient elevations are more consistently observed after parenteral than after oral introduction of the offending agent [38]. Applying the paired acute–baseline approach rather than a population threshold substantially improves detection, the “20% + 2” criterion achieving a sensitivity of 78% and a specificity of 91% [39,40]. The third is analytical interference. The assay is robust against the interferents commonly encountered in practice, such as rheumatoid factor or human anti-mouse antibodies at the concentrations usually present in patient samples. However, higher titres of heterophilic antibodies can produce a spuriously elevated result in patients who have neither anaphylaxis nor mastocytosis. Interference should therefore be suspected whenever a raised tryptase is discordant with the clinical picture, and it can usually be confirmed or excluded by repeating the measurement after blocking or on an alternative platform [41,42]. Finally, an elevated baseline value does not indicate its own cause. In fact, a raised BST is found in HαT, in chronic kidney disease, in myeloid neoplasms and in helminth infection, and treatment with glucocorticoids may further confound interpretation. Mastocytosis is among the least common of these explanations in unselected populations; a persistently elevated BST therefore identifies a patient who requires a differential diagnosis, not one who has a mast cell disorder [24,32,43]. Lastly, moderately raised tryptase has been reported in post-mortem samples from deaths of non-anaphylactic cause, so that post-mortem values require correspondingly cautious interpretation (ImmunoCAP™ Tryptase, Directions for Use 52-5467-EN/05; Phadia AB: Uppsala, Sweden, 2022).

### 3.5. Serum Tryptase Compared with Other Mediators of Mast Cell Activation

Serum tryptase remains the clinical reference standard for confirming systemic MC activation and for screening for underlying clonal disease, and no other marker matches its specificity (Table 1) [7,44]. Urinary metabolites of histamine, prostaglandin D_2_ (PGD_2_) and the cysteinyl leukotrienes capture localized reactions, remain informative when the acute blood draw is missed, and can guide targeted treatment. Histamine rises earlier than tryptase and in far larger molar amounts, but a plasma half-life of 1–2 min makes plasma sampling impractical. Its urinary metabolite N-methylhistamine, measured on a spot or 24 h collection, avoids this problem but is confounded by diet, by monoamine oxidase inhibitors, by histamine of bacterial or basophil origin, and by age-dependent reference ranges [45,46]. PGD_2_ is the most abundant prostanoid released by activated MC, and its stable urinary metabolites 11β-prostaglandin F2α and 2,3-dinor-11β-prostaglandin F2α are the markers most often found elevated in MCAS cohorts; they also identify patients with prostaglandin-predominant symptoms in whom aspirin therapy is appropriate. Two caveats apply: PGD_2_ is also produced by platelets, alveolar macrophages and Th2 lymphocytes, and its metabolites are suppressed by aspirin and other non-steroidal anti-inflammatory drugs, so recent treatment must be recorded [45,47]. Urinary leukotriene E4 is the least MC-specific, since cysteinyl leukotriene production is shared with basophils and eosinophils, but it is elevated in both clonal and non-clonal activation. It is useful for tracking respiratory and gastrointestinal flares, and leukotriene-modifying drugs and aspirin-exacerbated respiratory disease affect its interpretation [44,47]. For all three metabolites the comparison is with the patient’s own asymptomatic baseline, not with a population range. A rise of about 200% above baseline has been proposed as evidence of an activation event, although it is less firmly validated than the “20% + 2” equation [45,46].

Anaphylaxis deserves a separate comparison. Tryptase is highly specific for MC but only moderately sensitive, whereas platelet-activating factor (PAF)—released by MC, basophils, macrophages and platelets—tracks reaction severity more closely. In a landmark study PAF rose and PAF-acetylhydrolase activity fell in proportion to severity, and low PAF-acetylhydrolase activity was associated with fatal peanut anaphylaxis [48]. The finding has been reproduced in children, in whom PAF-acetylhydrolase outperformed tryptase as a marker of severe reactions. PAF measurement is not yet standardized for routine use, however, so that tryptase remains the most accessible rather than the most sensitive marker of anaphylaxis [49]. No single biomarker captures every presentation of MC activation. Tryptase remains irreplaceable for systemic anaphylaxis and for mastocytosis screening, but combining it with urinary metabolites potentially increases sensitivity in complex multisystem presentations, and current guidance recommends measuring the metabolites when tryptase is unavailable, inconclusive, or normal despite a convincing clinical picture [44,46,47].

**Table 1:** Comparison of serum tryptase with the other measurable mediators of mast cell activation.

Biomarker (Specimen)	Proposed Criterion	Main Advantages	Main Limitations	Reference
Total tryptase (serum)	Acute value ≥ 1.2 × baseline + 2 μg/L	Highest MC specificity; stable analyte with a half-life of approximately 2 h; widely available; tracks MC burden; validated within-patient criterion	Moderate sensitivity, missing mild or localized activation; narrow 30 min–4 h sampling window; requires two context-specific draws; does not distinguish burden from activation; raised in HαT, chronic kidney disease and myeloid neoplasms	[24,32,33,39,50]
Mature β-tryptase (serum)	—	Specific marker of degranulation rather than of MC burden	Not available outside research settings	[29,30,32]
Histamine (plasma)	—	Released in far larger molar amounts than tryptase and rises earlier; reflects immediate allergic and vasomotor symptoms	Half-life of 1–2 min, so sampling is almost never feasible in practice; also basophil-derived	[2,38,45]
N-methylhistamine (spot or 24-h urine)	Rise of ~200% above the individual baseline (proposed)	Overcomes the very short half-life of histamine; usable on a spot or timed collection	Confounded by dietary intake, MAO inhibitors, and histamine of bacterial or basophil origin; age-dependent reference ranges	[44,45,46]
11β-PGF2α and 2,3-dinor-11β-PGF2α (spot or 24-h urine)	Rise of ~200% above the individual baseline (proposed)	Stable metabolites of the most abundant mast cell prostanoid; the metabolites most frequently elevated in MCAS; guide the initiation of aspirin therapy	PGD_2_ is also produced by platelets, alveolar macrophages and Th2 cells; suppressed by aspirin and other NSAIDs; requires specialized handling; limited availability	[44,46,47]
Leukotriene E4 (spot or 24 h urine)	Elevation above the reference range	Elevated in both clonal and non-clonal mast cell activation; useful for tracking respiratory and gastrointestinal flares; increases panel sensitivity	Low mast cell specificity, being shared with basophils and eosinophils; altered by leukotriene-modifying drugs and by aspirin-exacerbated respiratory disease	[44,47]
PAF/PAF-acetylhydrolase (plasma)	—	Correlates with the severity of anaphylaxis better than tryptase; low PAF-acetylhydrolase activity is linked to fatal anaphylaxis	Not standardized for routine use; very short half-life; confined to research settings	[48,49]

HαT, hereditary α-tryptasemia; MAO, monoamine oxidase; MC, mast cell. MCAS, MC activation syndrome; NSAID, non-steroidal anti-inflammatory drug; PAF, platelet-activating factor; PGD_2_, prostaglandin D_2_; PGF2α, prostaglandin F2α. μg/L is numerically equivalent to ng/mL. The “20% + 2” criterion for tryptase is validated by international consensus, whereas the thresholds proposed for the urinary metabolites are less firmly established; for all of these markers the relevant comparison is with the patient’s own asymptomatic baseline rather than with a population reference range.

## 4. Recommendations from Current International Guidelines

Recommendations on the clinical use of tryptase are still fragmented across several organizations’ documents, which are complementary rather than competing, as each addresses a different clinical question. Overall, tryptase is a context-dependent biomarker, and the guidance for an acute event differs substantively from that for a persistently elevated baseline.

For anaphylaxis, European Academy of Allergy and Clinical Immunology (EAACI) guidelines recommend obtaining an acute sample within 1–2 h of symptom onset and a baseline sample at least 24 h after complete resolution, positioning serum tryptase as support for a clinical diagnosis rather than as a defining criterion [50]. The World Allergy Organization (WAO) guidance endorses that interpretation must integrate clinical findings with the comparison of acute against baseline values, and does not treat any single tryptase concentration as diagnostic in isolation [51,52]. Moreover, the 2023 American Academy of Allergy, Asthma & Immunology (AAAAI)/American College of Allergy, Asthma & Immunology (ACAAI) Joint Task Force practice parameter update adds an indication-based recommendation: baseline serum tryptase should be measured in patients with recurrent, idiopathic or severe anaphylaxis, in hymenoptera venom allergy, and where a MC disorder is suspected [53]. All three documents stress that the acute value must be interpreted against the individual baseline using the “20% + 2” equation rather than against a population reference limit, that a normal tryptase does not exclude anaphylaxis, and that treatment must never be delayed pending laboratory confirmation.

For MC disorders, the World Health Organization (WHO) classification and the European Competence Network on Mastocytosis (ECNM)/American Initiative in Mast Cell Diseases (AIM) consensus documents converge on a BST persistently above 20 μg/L as a minor diagnostic criterion for SM, with the explicit caveat that the value must be adjusted, or the criterion disregarded, in the presence of HαT or of an associated myeloid neoplasm [36,54]. The ECNM/AIM position is therefore sequential rather than absolute: the baseline value should first be interpreted within the biological reference interval of 1–15 μg/L, adjusted for the presence of HαT, and only then assessed against the WHO threshold [31]. For MCAS, both the consensus criteria and the AAAAI work group report require an event-related rise in tryptase above the individual baseline, and caution against diagnosing the condition from symptoms alone [44,46,55].

For hymenoptera venom allergy, European recommendations converge on measuring BST in every patient evaluated for a systemic sting reaction, since an elevated value identifies patients at higher risk of severe reactions, of side effects during immunotherapy and of relapse after its discontinuation, and supports the indication for lifelong venom immunotherapy in patients with mastocytosis or with an elevated baseline [56,57,58,59]. Current guidance also increasingly recommends tryptase genotyping in patients with elevated BST, particularly in the context of venom allergy, idiopathic anaphylaxis or suspected MC disease [13,31,58].

## 5. Causes of Elevated Serum Tryptase: Transient Versus Persistent

An elevated serum tryptase is a critical biomarker in MC disorders, hematological malignancies and severe allergic reactions, but it is not in itself a diagnosis. The cornerstone of the differential diagnosis is whether the elevation is transient or persistent (Table 2). A transient elevation returns to the individual’s baseline within 24–48 h and reflects acute degranulation with release of preformed mature tetramers, while a persistent elevation, confirmed on a sample taken in a symptom-free interval at least 24–48 h after any acute event, reflects constitutive secretion of inactive protryptase zymogens, and therefore indicates an increased MC burden, greater constitutive output per cell, or reduced clearance [32,60].

*Transient elevations*. Confirmation requires a rise fulfilling the consensus formula (acute tryptase ≥ 1.2 × baseline + 2 μg/L), not a value exceeding a population threshold. They occur in anaphylaxis of any etiology, IgE- or non-IgE-mediated, triggered by foods, drugs or insect venoms, although food-induced reactions frequently show a low or entirely normal profile. Acute MCAS flares produce the same pattern, causing multi-organ dysfunction without any increase in total MC mass. Medical interventions are a further important cause: general anesthesia, perioperative neuromuscular blocking agents and radiocontrast media all induce degranulation, through IgE- or MRGPRX2-mediated mechanisms [22,61].

*Persistent elevations*. HαT is the commonest cause: an autosomal dominant trait present in up to 6% of the population, caused by *TPSAB1* copy number gain [10,12]. In clonal MC neoplasms, a persistent BST above 20 μg/L is a minor diagnostic criterion for SM, and in highly proliferative variants such as MC leukemia values may exceed 200 μg/L [36,63]. Because MCs share a myeloid lineage, non-MC hematological disorders also raise tryptase through cross-lineage expression: acute myeloid leukemia, particularly core-binding factor leukemias with inv(16) or t(8;21), chronic myeloid leukemia, myelodysplastic syndromes and chronic eosinophilic leukemia [64]. Two non-hematological causes complete the differential: chronic kidney disease, where reduced clearance of protryptase produces elevations mirroring the fall in glomerular filtration rate [66], and severe helminth infection [2].

Population-level data have reordered the relative frequency of these causes, and the result contradicts long-standing clinical assumptions. Among subjects with BST ≥ 11.5 μg/L in a regional health system, nearly 90% had HαT, chronic kidney disease, a myeloid neoplasm or a combination of these, with HαT by far the commonest [24]. Mastocytosis, historically considered among the first diagnoses to exclude, is uncommon in unselected populations. Therefore, practically, in a patient with a moderately raised BST and no skin lesions, organomegaly or unexplained cytopenias, tryptase genotyping and assessment of renal function should precede any consideration of bone marrow examination [13].

## 6. Clinical Utility of Serum Tryptase in Different Settings

### 6.1. Tryptase and Anaphylaxis

**Figure 3:**
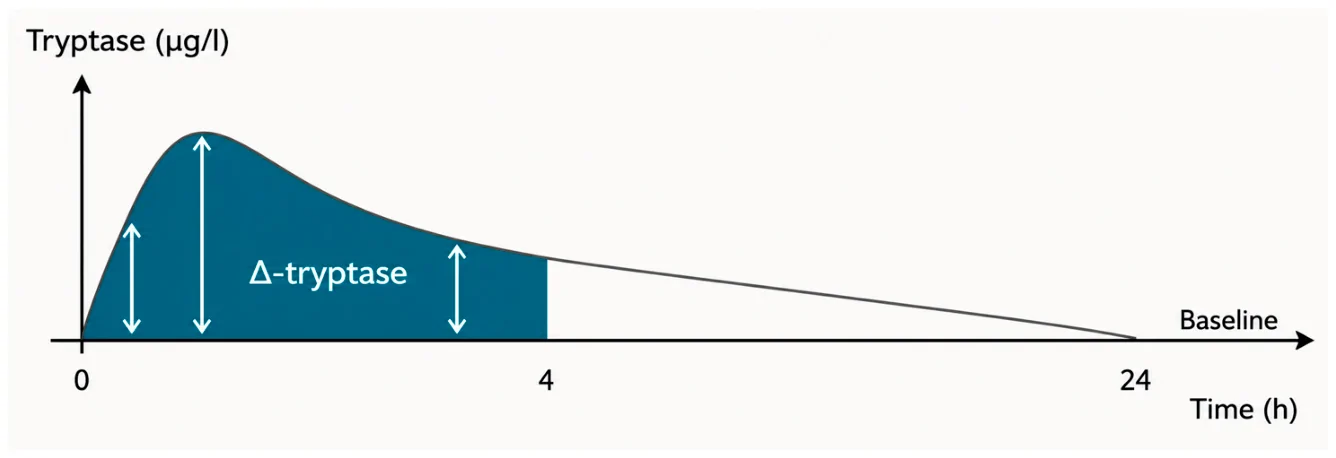
Time course of serum tryptase after systemic mast cell activation. Reproduced by courtesy of Thermo Fisher Scientific. Confirmed MC activation: if the increase in tryptase (Δ-tryptase) is at least 20% above baseline plus 2 μg/L. Reproduced by courtesy of Thermo Fisher Scientific.

Measurement of tryptase is useful for diagnostic purposes in the context of an anaphylactic reaction caused by different triggers such as hymenoptera venom, drug, latex, and food. Tryptase elevation is determined by the comparison of the level during the acute setting compared to the baseline level. Tryptase levels are transiently elevated in subjects with systemic anaphylaxis and, since it diffuses slower than the histamine, the tryptase level peaks between 15 and 120 min after the anaphylactic event with a degradation halftime of 2 h [33]. Usually, the elevated levels of tryptase can be detected for up to 3 to 6 h after the anaphylactic reaction and it returns to the pre-event baseline within 24–48 h, more slowly after severe reactions [3,33] (Figure 3). Interestingly, available evidence showed that more than 30% of patients with acute anaphylaxis did not exhibit an elevated tryptase level [67]. Five factors account for this: sampling outside the 30 min to 4 h window; comparison against a population reference limit instead of the patient’s own baseline, since a rise from 3 to 8 μg/L satisfies the “20% + 2” criterion while remaining within the normal range; the cellular source, food-induced reactions being driven largely by mucosal MCT and basophils, which contain far less tryptase; selective or piecemeal mediator release without substantial degranulation; and the contribution of non-MC mediators, PAF in particular. Tryptase is therefore a useful rule-in but a poor rule-out test, and anaphylaxis remains a clinical diagnosis. For patients with suspected anaphylaxis, identified guidelines suggest measuring tryptase levels 1 to 2 times during the event onset (e.g., immediately or within 4 h) and 1 to 2 times after resolution of symptoms (e.g., after 24 h) to establish baseline tryptase levels [67]. If there is a suspicion of mastocytosis or other conditions, such as hyper-tryptasemia, which are characterized by persistently elevated tryptase baseline values, a third measurement is usually carried out 1–2 weeks after the event. Notably, though tryptase is easy to measure and very specific to mast cells, the sensitivity to diagnose anaphylaxis is only around 70–80% [53].

Another context in which tryptase measurement is of help is in the post-mortem setting, taking the sample within 48 h of death. Elevated mast-cell-derived tryptase levels in postmortem sera reflect antemortem mast cell activation and may be used as a marker for fatal anaphylaxis. A post-mortem measurement on femoral blood is recommended to establish whether anaphylaxis was the cause of death. As a consequence, this parameter should be evaluated, both in the diagnosis of acute allergic reactions and in the management of allergic patients, particularly those with hymenoptera venom allergy, to guide the prescription of life-saving drugs, such as adrenaline auto-injectors, and intervention (i.e., allergen-specific immunotherapy) due to its prognostic role. To effectively use tryptase in the different clinical areas of application it is advisable not only to evaluate the absolute value with respect to the age cut-off, but to verify the trend over time and as a prognostic biomarker. This is believed to be the most clinically useful approach to treatment [68].

In addition to a diagnostic role, tryptase can be considered as a prognostic biomarker. A high baseline tryptase level is a risk marker for severe anaphylactic reactions since the baseline level of tryptase pro-forms reflects the number of MCs present in the subject. A baseline level above approximately 10 μg/L has traditionally been regarded as a marker of increased risk of severe reactions, particularly in patients with previous anaphylaxis. It should be stressed that this is a clinical decision threshold prompting further evaluation, and not an upper limit of normal, which is now set at 15 μg/L (Section 3.3). In hymenoptera venom allergy, a level > 6.4 μg/L has been found to predict the most severe grade of anaphylaxis, together with male sex and age over 60 [57,60,69]. Moreover, older age, male gender, arterial hypertension, and hypercholesterolemia, together with increased levels of BST ≥ 6.4 μg/L represent risk factors for severe anaphylaxis to hymenoptera venom in adults [70].

Considering drug (antibiotics, neuromuscular blocking agents, chlorhexidine, iodinated contrast media) and latex allergy, elevated BST levels indicate an increased MC burden, confirming MC degranulation regardless of the underlying pathophysiological mechanism (both IgE- and non-IgE-mediated) and may serve as a risk factor for severe reactions during surgery. To confirm an anaphylactic reaction, the importance of measuring the transient increase in tryptase during the perioperative phase is well established: a transient elevation immediately after the reaction indicates that it was MC-mediated. If positive, further allergy investigations, e.g., specific IgE tests, skin testing or provocation tests should be performed to find the likely trigger of the reaction [58,62,71].

Considering insect venom allergy (particularly hymenoptera venom allergy), tryptase helps to confirm the diagnosis of an anaphylactic reaction and to stratify risk, an elevated value identifying a higher risk of treatment failure and of relapse, including fatal reactions, after venom immunotherapy is stopped. Moreover, tryptase measurement guides treatment, with the indication of a prolonged venom immunotherapy in all patients with mastocytosis and/or BST > 11.4 µg/L [57]. Furthermore, patients with recurrent anaphylaxis with a nonidentifiable trigger present a particular challenge in diagnosis and management. In these patients, the possibility of clonal disease should be considered, and may be suggested by hypotensive episodes with urticaria and angioedema, and high BST [72].

### 6.2. Mast Cell Activation Syndrome (MCAS)

MCAS denotes a condition in which episodic, recurrent signs and symptoms of systemic MC mediator release affect at least two organ systems, with no sufficient alternative explanation. The consensus criteria require three elements concurrently: typical episodic multisystem symptoms, objective evidence of MC involvement (established by a rise in serum tryptase above the patient’s own baseline according to the “20% + 2” equation during a symptomatic episode), and a documented response to drugs targeting MC, their mediators, or mediator receptors. Most misapplications of the MCAS criteria concern the biochemical one, which is the criterion that decides whether the diagnosis holds. The “20% + 2” rule is a within-patient comparison: severe systemic MC activation is marked by a reversible rise above the individual’s own baseline, not by any absolute concentration. A single high value therefore does not establish the diagnosis, and a normal value does not exclude an episode sampled outside the diagnostic window. This matters, because MCAS is increasingly misdiagnosed in patients with chronic, non-episodic complaints in whom mediator release was never documented [44,46,50,56,61].

Three etiological subtypes are recognized. Primary (clonal) MCAS arises from a clonal MC population and is defined by *KIT* D816V and/or aberrant CD25 expression, either within established SM or as monoclonal MC activation syndrome, where clonality markers are present but the full SM criteria are not met. Secondary MCAS is driven by an identifiable external trigger, typically IgE-mediated allergy, with hymenoptera venom allergy as the paradigm. Idiopathic MCAS is diagnosed when the criteria are met but neither clonality nor a trigger can be demonstrated [56,72,73].

One point connects MCAS to themes developed earlier. HαT is substantially enriched among patients investigated for MC activation symptoms and raises baseline tryptase without constituting clonal disease; failing to recognize it leads both to unnecessary bone marrow examination and to misattributing a raised baseline to MCAS [11,13].

### 6.3. Systemic Mastocytosis

The persistence of high baseline levels in the blood reflects a pathological increase in the mast cells and immature myeloid cells’ burden in the body, as it happens in conditions like systemic and cutaneous mastocytosis in systemic evolution, hereditary α-tryptasemia (HαT) and hematological disorders. In well-defined clinical settings a persistently high baseline tryptase level (>20 μg/L) is frequently associated with SM [74]. The World Health Organization (WHO) recommends the determination of “Total Tryptase” as a minor diagnostic criterion in the diagnosis of SM (Table 3) [36].

According to the WHO classification [36,55,75,76], SM is divided into categories of differing severity, and a tryptase value > 200 μg/L constitutes a B-finding; in most cases the basal levels of tryptase correlate with the severity of the disease and therefore represent a valid aid in monitoring the disease and the efficacy of cytoreductive therapy [61]. Clinically, SM can be further divided into bone marrow mastocytosis (BMM), which was reclassified as a discrete SM subtype in the 2022 WHO classification; indolent; smoldering; and advanced. The non-advanced subtypes are by far the most frequent and comprise BMM, indolent SM (ISM) and smoldering SM (SSM), whereas advanced SM comprises aggressive SM (ASM), SM with an associated haematologic neoplasm (SM-AHN) and mast cell leukemia (MCL) [76,77]. SM is an increased risk factor for anaphylactic reactions, particularly in response to hymenoptera bites or medications [56,78]. Of note, in a phase 1 trial of the MC silencer lirentelimab in refractory indolent SM, patients receiving single or multiple doses of lirentelimab did not meaningfully reduce BST levels despite a significant clinical improvement [79].

HαT, described in Section 2.4, is a further and far more common cause of an elevated BST, and the two may coexist. As discussed in the consensus proposal of Valent et al. [36], the tryptase level should be adjusted in a patient with known HαT, although the optimal method of adjustment remains to be defined. HαT has been consistently associated with clonal MC disorders and, among these patients, with more frequent and more severe anaphylaxis to hymenoptera venom, as well as with idiopathic anaphylaxis [73]. Individuals with HαT are also at higher risk of severe food allergic reactions, so that tryptase genotyping may serve as a biomarker of food allergy severity [16]. BST should therefore be measured in every patient with severe food-induced reactions, idiopathic anaphylaxis or venom allergy [73]. An increased relative number of α-tryptase-encoding gene copies, even in the absence of HαT, has also been associated with SM and has been shown to positively correlate with the severity of MC-mediated reactions to vibration and food. An increased generation of α/β-tryptase heterotetramers and differences in their enzymatic activity relative to β-tryptase homotetramers may explain these findings [14,17,26,61,80].

The severity of the symptoms and the number of organs involved are extremely variable. Gastrointestinal symptoms, neuropsychiatric symptoms, allergic and pseudoallergic cutaneous and systemic reactions, musculoskeletal pain, symptoms of autonomic vascular dysfunction, and connective tissue anomalies have been reported [17,26].

Interpretation of the baseline value in this setting must start from the reference interval of 1–15 μg/L defined in asymptomatic controls, HαT carriers included (Section 3.3) [31]. Genotyping should be performed when BST exceeds 11 μg/L, particularly in hymenoptera venom allergy [59]. Chovanec et al. have developed a BST calculator that defines patient-specific reference ranges from the *TPSAB1* replication number [28]. Polivka et al. have reported a significantly higher prevalence of HαT in all subtypes of SM than in controls, suggesting that HαT is both a pathophysiological contributor to, and a risk factor for, mastocytosis [27].

### 6.4. Cutaneous Mastocytosis

Currently, the World Health Organization (WHO) recognizes three major variants of cutaneous mastocytosis (CM): maculopapular cutaneous mastocytosis (MPCM), diffuse cutaneous mastocytosis (DCM), and solitary mastocytoma [55,76,81,82].

In about half of the adults with mastocytosis, the diagnosis is made because of their characteristic monomorphic skin lesions of maculopapular cutaneous mastocytosis, also called Urticaria Pigmentosa (MPCM/UP). In contrast to pediatric mastocytosis, where lesions may vary between different children in terms of size, pigmentation, induration and elevation, having been termed “polymorphic”, lesions in adults are typically small 3–4 mm red-brown macules or slightly elevated monomorphic papules (duck skin) [83].

It has been reported that BST levels > 20 μg/L in CM reflect either extensive skin involvement or the possibility of systemic disease, particularly in pediatric mastocytosis [84]. A BST exceeding 20 μg/L furthermore remains a minor SM criterion, with the caveat that HαT and myeloid neoplasms may themselves raise tryptase. All patients with BST > 20 µg/L should be referred to a hematologist for further diagnostic examination [58].

In the majority of children with cutaneous mastocytosis, including most of those with MPCM/UP, BST lies within the normal range; a normal value therefore neither excludes nor argues against the diagnosis, which rests on the characteristic cutaneous findings. Elevated values identify a minority with extensive skin involvement. Children with MPCM usually experience MC mediator-related symptoms, which are more prominent when the skin lesions are extensive. Children with familial DCM often show persistently increased BST levels, while a chronic course of the disease and mast cell infiltration in extracutaneous organs occur in some. Children with extensive skin lesions and elevated BST levels may also suffer from cardiovascular symptoms, including tachycardia, hypotension, collapse and shock [85].

BST levels generally decrease with time in pediatric patients with mastocytosis and are a good biomarker for resolution. The most common mutation of the c-KIT gene [tyrosine-protein kinase KIT or cluster of differentiation 117 (CD117) or mast/stem cell growth factor receptor (SCFR)] in MC neoplasms is D816V. Its diagnostic weight differs markedly with age: D816V is detectable in more than 90% of adults with SM, but in only a minority of children, in whom mutations frequently involve exons 8, 9 and 11 outside codon 816 and a proportion of cases carry no detectable KIT mutation. A negative D816V result in a child therefore has little diagnostic value and does not argue against mastocytosis. Whereas baseline levels of serum tryptase (BST) levels ≥ 20 μg/L together with severe mediator symptoms alone are no indication for a bone marrow (BM) biopsy, organomegaly or demonstration of the c-KIT D816V mutation may indicate SM and also an increase in BST over time, suggesting the need for monitoring over concern about disease progression. Where the marrow is sampled, flow cytometric immunophenotyping (CD2, CD25, CD30, CD117) of the aspirate may establish a minor WHO criterion without recourse to histology [86].

In adults with skin lesions who did not yet undergo a complete staging with BM analyses, the provisional diagnosis of “mastocytosis in the skin” (MIS) is appropriate. In children, the provisional diagnosis of MIS does not apply unless serum tryptase levels exceed 100 μg/L and/or clear signs for a systemic hematologic disease (e.g., unexplained splenomegaly) are found and no BM studies were performed. Finally, a score of the clinical extent of mastocytosis, the SCORMA (SCORing MAstocytosis) Index, was developed in 2009. The SCORMA Index and serum tryptase have been shown to correlate moderately, indicating that both are valuable for assessing CM or SM in cases where the skin is involved [87].

### 6.5. Chronic Spontaneous Urticaria

CSU is defined by the spontaneous appearance of widespread wheals, angioedema, or both, daily or on most days of the week for at least 6 weeks, with an unpredictable course of varying frequency and severity. MC activation is central to its pathogenesis, MC releasing a range of mediators including histamine and tryptase [88]. CSU is a relatively frequent pathology, with a prevalence in the general population between 0.4% and 1% and characterized by a strong significant impairment to the patient’s quality of life due to the persistence, even for years, of symptoms such as itching, wheals and angioedema [89]. According to the most recent studies about 40% of cases of CSU are caused by a type II b autoimmunity: in these patients, the autoantibodies are responsible for the activation and degranulation of mast cells and basophils [90,91]. There is no specific biomarker to evaluate the activity of CSU and the suitability of tryptase measurement within a panel of tests for assessing disease activity is debated [56]. Therefore, it is currently performed only for study/research purposes. BST does tend to be higher than in healthy subjects, but for the reasons set out below this reflects MC number rather than systemic MC activation [91]. Overall, BST is not useful for diagnosing localized MC activation [92]. Tryptase measurement should nonetheless be considered in the differential diagnosis with other conditions, in particular mastocytosis [93].

The apparent paradox deserves explicit comment, since MC degranulation is unquestionably central to the pathogenesis of CSU and histamine is demonstrably released within the skin [91]. Tryptase, in fact, is released locally into the dermis alongside histamine in autoimmune urticaria: the discrepancy therefore lies in the detection rather than in the release, and it is attributable to the physical containment of the mediator, to the identity and number of the cells activated, and to the strictly localized nature of the immune response [94]. The activation is spatially confined to cutaneous MC, and the total mass of MC involved is a very small fraction of the body burden, so that any tryptase released is diluted into the systemic circulation far below the threshold of detection. Moreover, tryptase is a large tetramer that reaches the circulation slowly by lymphatic drainage rather than direct vascular absorption, dissociating into inactive monomers *en route*. By contrast, histamine is released locally and acts at once on adjacent vessels and nerve endings to produce the visible lesion, without ever needing to reach the systemic circulation in measurable amounts [2,7]. In addition, CSU is characterized by chronic low-grade and often piecemeal MC activation rather than by the massive synchronous degranulation that characterizes systemic anaphylaxis [91].

Consistently, the modestly higher total tryptase reported in patients with active chronic urticaria is not accompanied by a rise in mature β-tryptase, indicating that it reflects an increased MC number or increased constitutive output rather than systemic degranulation [92]. In unselected CSU cohorts, baseline values show wide variability with limited correlation to disease severity, so that tryptase performs poorly as a marker of disease activity [93]. The same reasoning applies to localized allergic reactions in general: rhinitis, conjunctivitis, isolated urticaria and localized angioedema are not accompanied by measurable serum tryptase elevation, and tryptase should not be requested as a diagnostic test in these settings [2].

Finally, it is of interest that serum tryptase is also not useful in assessing the pharmacodynamic effect of agents that block MC activation without altering MC count, like omalizumab (anti-IgE antibody) and therefore not useful for predicting treatment response to it [95].

### 6.6. Hematological Disorders

Overall, in hematological malignancies (acute myeloid leukemia, chronic myeloid leukemia, chronic eosinophilic leukemia) tryptase represents a diagnostic and prognostic biomarker [58]. BST may be elevated in hematological disorders caused by the uncontrolled growth of immature myeloid cells in the bone marrow. The proportion of patients with a raised value varies with the disease, from 20–30% in myelodysplastic syndrome to 40–50% in chronic myeloid leukemia and 40–60% in chronic myelomonocytic leukemia. In all these cases, an associated SM should be excluded. Monitoring this parameter during chemotherapy treatment proved to be a useful tool in the evaluation of the chosen drug, also leading to changes during the therapy itself [96].

Conversely, almost 20% of all patients with SM have been diagnosed with an associated clonal hematologic non-mast cell disease, mostly myeloid neoplasm. Patients with SM can also develop a primary eosinophilic disorder, i.e., chronic eosinophilic leukemia (CEL) and hypereosinophilic syndrome (HES), while in other cases, marked eosinophilia is demonstrable, but without fulfillment of the criteria for HES or CEL. An elevated BST does not count as an SM criterion in patients with a non-MC-lineage hematologic neoplasm. Interestingly, an elevated serum tryptase level not only points to MC-lineage involvement in primary eosinophilic disorders but may predict responses to imatinib [65]. Patients with eosinophil-related myeloid neoplasms with rearranged PDGFR fusion genes may also have increased basal serum tryptase levels [14].

### 6.7. Chronic Kidney Disease

Impaired renal function is among the most frequent and most consistently overlooked non-clonal causes of a persistently raised BST, deserving systematic consideration in any patient investigated for elevated tryptase. Serum tryptase rises progressively as renal function declines, correlating inversely with the estimated glomerular filtration rate (eGFR) and directly with markers of renal dysfunction, and the highest values are observed in advanced chronic kidney disease (CKD) and in end-stage renal disease (ESRD) [66]. In the population-level analysis, CKD ranked immediately after HαT among the identifiable causes of BST ≥ 11.5 μg/L [24], and it is explicitly listed among the conditions that confound the interpretation of baseline values [31].

Several mechanisms have been proposed, which illustrate a more general interpretative problem. A raised total tryptase may reflect an increased MC burden, ongoing activation, increased constitutive transcription or reduced clearance, and current assays cannot distinguish between them [32]. Reduced renal clearance of constitutively secreted proforms is likely to contribute, but it is probably not the whole explanation, since MCs accumulate within the fibrotic kidney, where tryptase promotes fibroblast proliferation and extracellular matrix deposition through PAR2 signaling contributing to non-classical activation of the renin-angiotensin system. Consistent with an active pathogenic role, higher serum tryptase concentrations have been reported to associate independently with progression to ESRD and with mortality in advanced CKD [66].

Two practical implications follow. First, renal function should be documented before any invasive work-up is contemplated: in a patient with significant renal impairment, a raised BST is not by itself evidence of a clonal MC disorder, and treating it as such is precisely the overinterpretation that the revision of the reference interval was meant to prevent [24,31]. Second, when a patient with CKD requires investigation for suspected anaphylaxis, the paired acute-versus-baseline approach matters more than ever, since the population reference interval is uninformative in this group and only the patient’s own baseline offers a meaningful comparator [32].

### 6.8. Other Clinical Settings

Considering other allergic manifestations such as food allergy, Licari et al. found significantly higher values of serum tryptase after oral food challenge in patients with positive response, compared to those with negative ones, even if these values are far below the established pathologic cut-off. No anaphylactic reactions were observed in the positive response group, apparently in contrast with previous studies, which reported a lack of tryptase elevation in food-induced anaphylaxis [97].

In cardiovascular diseases (atherosclerosis, acute coronary syndrome) tryptase may serve as a biomarker of advanced peripheral atherosclerosis and development of aneurysms and a possible long-term prognostic biomarker [58].

Tryptase measurement is also of use in gastrointestinal diseases: in irritable bowel syndrome it can confirm the diagnosis and higher BST levels are observed in IBS patients. Moreover, eosinophilic esophagitis patients with significantly increased BST levels suggest diffuse diseases like eosinophilic gastroenteritis and extraesophageal symptoms. In inflammatory bowel disease tryptase is considered a biomarker of fibrosis and an indicator of the extra intestinal manifestations. Among other malignancies, like hepatocellular carcinoma, it correlates with tumor angiogenesis and it is useful for monitoring the response to treatment [58]. Moreover, tryptase represents one of the main proangiogenic non-classical factors acting in melanoma, unfortunately without a current clinical reflection [98]. Tryptase levels may also increase during chronic infections associated with MC hyperplasia [14]. In fact in parasitic infections (*Trypanosoma* species, *Toxoplasma gondii*, *Plasmodium* species, *Leishmania* species, *Onchocerciasis*) tryptase has shown its utility as a potential diagnostic and monitoring biomarker [58].

Finally, there is an emerging role for serum tryptase in asthma endotyping [99]. In a post hoc analysis of phase III clinical trial of omalizumab in asthma, it was shown that patients who lacked α-tryptase—and conversely, have more active β-tryptase-containing alleles—presented with Th2 low asthma. This specific type of asthma was poorly responsive to omalizumab therapy and was linked to higher levels of mature tryptase in the respiratory tract [23].

## 7. Towards Personalized Management

Personalization here follows the same logic that has reshaped the classification of allergic disease more broadly: the recent EAACI position paper recasts allergic and hypersensitivity disorders on mechanistic and endotype-driven grounds, and tryptase interpretation belongs within that framework, since an identical numerical value means something different depending on whether it reflects IgE-mediated activation, MRGPRX2 engagement, a constitutional genetic trait or clonal proliferation [100]. The most fundamental shift is the replacement of a fixed population threshold by the individual’s own baseline, avoiding overdiagnosis in those whose constitutionally high baseline would otherwise be misread as an acute event [34,39,50]. Genotyping adds a second dimension, yielding a genotype-specific reference range, formalized in the BST calculator model, that distinguishes a constitutionally elevated baseline from a pathologically elevated one, avoiding invasive investigation where a raised BST is fully explained by HαT, while flagging patients whose value exceeds what their genotype predicts and in whom another cause should be sought [13,28]. Moreover, an elevated BST identifies patients at increased risk of severe anaphylaxis, particularly to hymenoptera venom and parenteral drugs, informing concrete decisions: adrenaline auto-injector prescription, the indication for and duration of venom immunotherapy, perioperative precautions, and the threshold for hematological referral [57,60,70]. In children with cutaneous mastocytosis, serial measurement guides follow-up intensity and the decision to investigate systemic involvement, while declining values reassure about spontaneous resolution [86,87]. In SM, BST tracks MC burden and contributes to monitoring disease course and response to cytoreductive therapy [63,74]. Finally, knowing when not to measure tryptase matters just as much. It adds little in localized allergic disease, as an activity marker in CSU, or in monitoring agents such as omalizumab, which inhibit MC activation without reducing MC number. Confining the test to settings where it changes management is itself individualized care [93,95].

## 8. Future Perspectives

Currently, the most immediate need is for assays that discriminate α- from β-tryptase, and mature enzyme from proforms, in routine practice: a solitary elevated total tryptase cannot distinguish increased MC burden from increased constitutive transcription or acute degranulation. Isoform-resolved assays, including those detecting α/β heterotetramers, would resolve much of this ambiguity and are technically within reach [7,13,14]. Harmonization is also a priority. Terminologically, the EAACI nomenclature paper provides a mechanistically grounded vocabulary that should be adopted consistently, since inconsistent use of terms such as anaphylaxis, hypersensitivity and MC has hampered comparison of tryptase data across cohorts [100]. Analytically, the revised 1–15 μg/L range has not been uniformly adopted and coexists with thresholds derived under the earlier convention. Prospective validation of genotype-adjusted reference ranges, and agreement on how values should be corrected in established HαT are necessary steps [28,31]. Pediatric values need particular attention, since age- and sex-specific thresholds await confirmation in larger cohorts [37]. Furthermore, tryptase should increasingly be read within a panel, in combination with urinary mediator metabolites, which improve the diagnostic yield in MCAS. Standardizing PAF and PAF-acetylhydrolase measurement would likewise address the sensitivity gap that tryptase alone cannot close [47,48,49].

Finally, tryptase is moving from a biomarker to a therapeutic target. Allosteric anti-tryptase antibodies have shown activity, and KIT inhibitors and MC silencers are entering clinical use, but agents that inhibit activation without reducing MC number may improve symptoms while leaving BST unchanged. Therefore, tryptase may prove an inadequate surrogate of response, and dedicated pharmacodynamic biomarkers will be needed [23,79].

## 9. Conclusions

Tryptase plays a crucial role as a biomarker for MC activation and proliferation, with significant implications in various clinical settings. Its measurement provides valuable diagnostic and prognostic insights, helping differentiate between allergic reactions and other systemic conditions. Despite its widespread use, limitations exist in specificity and interpretation, particularly concerning baseline levels influenced by genetic factors. Understanding the kinetics of tryptase release is essential for optimizing diagnostic accuracy, and elevated tryptase levels serve as a risk marker for severe anaphylactic events, guiding therapeutic decisions. In hematological diseases, persistent tryptase elevation suggests an increased MC burden, necessitating further investigation into conditions such as systemic mastocytosis. Future research should focus on developing more precise assays to distinguish between α- and β-tryptase isoforms and exploring novel therapeutic interventions targeting MC-mediated disorders. As our understanding of tryptase biology evolves, its clinical applications will continue to expand, improving patient outcomes through more personalized and accurate diagnostics.

## Figures and Tables

**Table 2:** Transient and persistent elevations of serum tryptase: mechanisms, causes and the features that distinguish them in clinical practice.

Pattern	Cause	Distinguishing Features	Reference
Transient elevation Acute degranulation with release of preformed, enzymatically active mature tryptase tetramers. Peaks at 15–120 min and normalization within 24–48 h. Confirmed by an acute value ≥ 1.2 × baseline + 2 μg/L. Marker of immediate systemic hypersensitivity or acute MC activation.	Anaphylaxis of any etiology (food, drug, hymenoptera venom, latex, idiopathic)	Rise fulfills the “20% + 2” equation above the individual baseline; peak 15–120 min; sampling window 30 min–4 h; food-induced reactions frequently show a normal profile	[33,38,50,61]
	Perioperative hypersensitivity (IgE- or MRGPRX2-mediated)	General anesthesia, neuromuscular blocking agents, radiocontrast media; paired acute and baseline samples essential; elevation confirms mast cell involvement irrespective of mechanism	[22,39,40,62]
	Acute flare of MCAS	Episodic, recurrent, multi-organ dysfunction without increased total mast cell mass; requires a documented event-related rise plus response to mast cell-directed therapy	[44,44,46]
	Envenomation	Direct physical or biochemical activation, typically by hymenoptera stings	[57,59]
Persistent elevation Constitutive secretion of inactive protryptase zymogens, reflecting an increased MC burden, an increased constitutive output per cell, or reduced clearance. Constantly elevated across repeated determinations obtained ≥24–48 h from any acute event. Marker of genetic traits, organ failure or clonal disease.	HαT	Commonest cause overall (up to 6% of the population); autosomal dominant; *TPSAB1* copy number gain with a gene-dose effect; may be entirely asymptomatic	[10,11,13]
	Systemic mastocytosis and other clonal MC disorders	Baseline > 20 μg/L is a minor WHO criterion; *KIT* D816V; aberrant CD25/CD2/CD30 expression; mast cell leukemia may exceed 200 μg/L	[36,55,63]
	Non-MC myeloid neoplasms (core-binding factor AML, CML, MDS, CEL, PDGFR-rearranged neoplasms)	Cross-lineage expression from a shared myeloid lineage; cytopenias or abnormal blood counts; an elevated baseline does not count as an SM criterion in this context	[64,65]
	CKD and ESRD	Reduced renal clearance of constitutively secreted protryptase; correlates inversely with glomerular filtration rate; second commonest identifiable cause	[24,66]
	Severe helminth infection and chronic parasitic disease	Prolonged reactive immune response; eosinophilia; relevant travel or exposure history	[2,58]
	Analytical interference (heterophilic antibodies)	Discordance between the tryptase value and the clinical picture; resolved by repeat testing after blocking or on an alternative platform	[41,42]

The biological reference interval for baseline serum tryptase in asymptomatic subjects, inclusive of carriers of HαT, is 1–15 μg/L; μg/L is numerically equivalent to ng/mL. AML, acute myeloid leukemia; CEL, chronic eosinophilic leukemia; CKD, chronic kidney disease; CML, chronic myeloid leukemia; ESRD, end-stage renal disease; HαT, Hereditary α-tryptasemia; MC, mast cell; MCAS, mast cell activation syndrome; MDS, myelodysplastic syndrome; MRGPRX2, Mas-related G protein-coupled receptor X2; PDGFR, platelet-derived growth factor receptor; SM, systemic mastocytosis; WHO, World Health Organization.

**Table 3:** Diagnostic criteria for systemic mastocytosis according to the 2022 WHO classification, showing the position of baseline serum tryptase among the minor criteria [36]. For diagnosis of SM, at least one major and one minor or three minor criteria are required.

Major criterion: Multifocal dense infiltrates of mast cells (≥15 mast cells in aggregates) detected in sections of the bone marrow and/or other extracutaneous organ(s).
Minor criteria: (a) >25% of all mast cells are atypical cells (type I or type II) on bone marrow smears or are spindle-shaped in dense and diffuse mast cell infiltrates in BM or other extracutaneous organ(s). (b) Activating *KIT* point mutation(s) at codon 816 or in other critical regions of *KIT* in the bone marrow or other extracutaneous organ(s). (c) Mast cells in bone marrow, blood, or other extracutaneous organ(s) aberrantly express one or more of the following antigens: CD2, CD25, CD30. (d) Baseline serum tryptase concentration >20 μg/L in the absence of a myeloid AHN. In the case of a known HαT, the tryptase level should be adjusted.

## Data Availability

No new data were created or analyzed in this study. Data sharing is not applicable to this article.
